# Elevated dry-season malaria prevalence associated with fine-scale spatial patterns of environmental risk: a case–control study of children in rural Malawi

**DOI:** 10.1186/1475-2875-12-407

**Published:** 2013-11-11

**Authors:** Lindsay R Townes, Dyson Mwandama, Don P Mathanga, Mark L Wilson

**Affiliations:** 1 Department of Epidemiology, School of Public Health, University of Michigan, 48104 Ann Arbor, MI, USA; 2 Malaria Alert Centre, College of Medicine, University of Malawi, Blantyre, Malawi; 3 Department of Community Health, College of Medicine, University of Malawi, Blantyre, Malawi

## Abstract

**Background:**

Understanding the role of local environmental risk factors for malaria in holo-endemic, poverty-stricken settings will be critical to more effectively implement- interventions aimed at eventual elimination. Household-level environmental drivers of malaria risk during the dry season were investigated in rural southern Malawi among children < five years old in two neighbouring rural Traditional Authority (TA) regions dominated by small-scale agriculture.

**Methods:**

Ten villages were randomly selected from TA Sitola (n = 6) and Nsamala (n = 4). Within each village, during June to August 2011, a census was conducted of all households with children under-five and recorded their locations with a geographic position system (GPS) device. At each participating house, a nurse administered a malaria rapid diagnostic test (RDT) to children under five years of age, and a questionnaire to parents. Environmental data were collected for each house, including land cover within 50-m radius. Variables found to be significantly associated with *P. falciparum* infection status in bivariate analysis were included in generalized linear models, including multivariate logistic regression (MLR) and multi-level multivariate logistic regression (MLLR). Spatial clustering of RDT status, environmental factors, and Pearson residuals from MLR and MLLR were analysed using the Getis-Ord Gi* statistic.

**Results:**

Of 390 children enrolled from six villages in Sitola (n = 162) and four villages in Nsamala (n = 228), 45.6% tested positive (n = 178) for *Plasmodium* infection by RDT. The MLLR modelled the statistical relationship of *Plasmodium* positives and household proximity to agriculture (<25-m radius), controlling for the child sex and age (in months), bed net ownership, elevation, and random effects intercepts for village and TA-level unmeasured factors. After controlling for area affects in MLLR, proximity to active agriculture remained a significant predictor of positive RDT result (OR 2.80, 95% CI 1.41-5.55). Mapping of Pearson residuals from MLR showed significant clustering (Gi* z > 2.58, p < 0.01) predominantly within TA Sitola, while residuals from MLLR showed no such clustering.

**Conclusion:**

This study provides evidence for significant, dry-season heterogeneity of malaria prevalence strongly linked to peridomestic land use, and particularly of elevated risk associated with nearby crop production.

## Background

Malaria represents a critical public health issue in a severely resource-constrained country such as Malawi. Nationally, clinically diagnosed cases of malaria increased from ~3.8 million in 2006 to over six million in 2009 [1]. Young children in Malawi are particularly burdened, averaging over one case per year, with little change in prevalence of malaria from 2001 to 2010 [1,2]. However, a recent nationwide survey indicates only 28% of under-five year olds were parasitaemic by microscopy [3]. All-cause mortality among under-five year olds in Malawi was 112 per 1,000, 15-40% of which is attributable to malaria [4,5]. In Machinga District, Malawi, the study area of this report, 72,155 new malaria cases were reported during 2004–2005 among under-five year old residents, who numbered 72,354, representing an average of one case per child per year [6]. More recent surveys indicate that malaria prevalence in Machinga District during the early dry season among children under age five was 43%, while 37% were found to be parasitaemic by PCR [7].

Although malaria is considered endemic throughout Malawi, spatial heterogeneity of transmission is not well characterized. Regional prevalence of fever among children under-five varies from 29% in the urban and peri-urban Blantyre to 47% in the highly rural Thyolo, while district-level incidence of under-five malaria varies similarly, from 997 per 1,000 in Machinga, to 715,636, and 542 in adjacent Zomba, Mangochi, and Balaka Districts, respectively. With as much as two-fold difference in incidence between adjacent districts, it is likely that finer scale analysis of environmental heterogeneity will show even greater geographic variability of malaria risk. Urban–rural differences in symptomatic malaria are also present, with rural area under-fives more often experiencing fever within the previous two weeks than did those from urban settings [3,6]. Despite important geographic variation in the epidemiology of malaria in Malawi, most studies have focused on socio-economic risk factors for the disease, while spatial, environmental and seasonal data are seldom explicitly included [1,8].

Diverse environmental exposures modulate patterns of malaria risk, particularly in rural areas, including land cover, agriculture, elevation, temperature, soil moisture, topography, human and vector population densities, and construction of human dwellings [9]. Some spatially explicit studies in other countries have explored these associations at relatively coarse geographic scales (i.e., hundreds of metres or more) to describe broad ecological and geographic patterns of malaria epidemiology [10-14]. However, malaria risk factors can vary tremendously at local or fine scale (i e, tens of metres or less), and these microgeographic patterns are increasingly being studied in order to more efficiently target anti-malaria interventions [15-20]. Of particular interest is how such local-scale environmental features contribute directly to variation in *Plasmodium* transmission, while interacting with sociocultural factors commonly associated with malaria disease [21,22].

This study was designed to investigate relationships among local land use/land cover, social factors and recent *Plasmodium falciparum* infection in children under five years of age around rural Liwonde, Malawi. These associations were investigated during the dry season when transmission was supposed to be less intense. In particular, this study’s aims included: 1) describe household-scale land cover and environmental risk factors for malaria, and evaluate associations with questionnaire-based demographic, socio-economic status (SES) and malaria knowledge, attitudes and practices (KAP); 2) identify clusters of environmental and social variation, at different spatial scales, that represent increased or decreased malaria risk; and, 3) generate new insights to aid decision-making on malaria risk management and mitigation.

## Methods

### Study site

**Figure 1:**
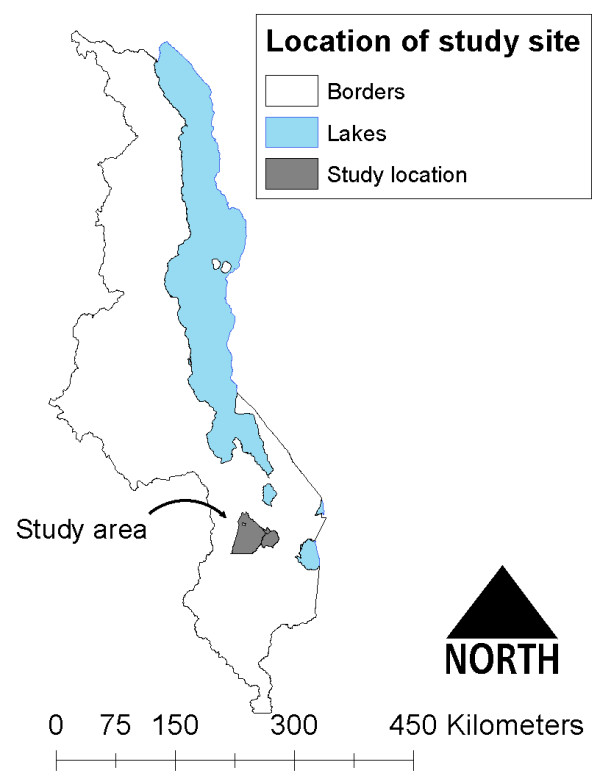
Location of study Traditional Authorities in Malawi.

This study was conducted within the catchment area of Machinga District Hospital (MDH), a government-operated hospital which receives patients from villages in Balaka and Machinga Districts along the Shire River of southern Malawi. In addition to the government-operated MDH, smaller private clinics are available in the region, but are beyond the financial means of most residents. Villages in two adjacent Traditional Authorities (TAs), Nsamala (pop. ~175,000) and Sitola (pop. ~38,000), were studied (Figure 1). Roughly 95% of the populations in both TAs are considered to be residents of rural settings, with >15% of residents under the age of five years [23]. The terrain is hilly, ranging from ~400 to ~750 m above sea level. The primary economic activity in the two TAs is farming, both subsistence and cash crops. The climate is semi-tropical, marked by wet-dry seasonality. In contrast to the rainy, wet season (November to April), this study occurred over the cool, dry season from June to August, when average temperatures range from 14°C to 28°C, with almost no precipitation [24]. While no direct survey of malaria vectors has been conducted in the Upper Shire Valley, where this study took place, malaria vectors in the Lower Shire Valley have been surveyed, and consist of *Anopheles funestus*, *Anopheles gambiae sensu stricto*, and *Anopheles arabiensis*[25]. These vectors are common throughout the country, and are likely the primary vectors in the Upper Shire Valley as well, as evidenced by a study of insecticide resistance around Liwonde National Park [26].

### Study participants

Ten villages were selected at random from the two TAs that surround Liwonde, Malawi (15.05°S; 35.22°E) using lists provided by each TA chief. Because of smaller village populations, six villages were selected from TA Sitola, and four from TA Nsamala. Additionally, because TA Nsamala is partly serviced by another district hospital, the village sampling frame was limited to those villages identified by the TA chief as primarily accessing health services at MDH. To conform with Malawian culture and to maximize participation, the research team met with the heads of the two TAs and ten villages, and received permission to work within their domains. The TA and village heads then discussed the study with the residents before the arrival of researchers. Using residents suggested by the village chiefs as guides, a census was conducted of all households with children under five years within each village. For households with more than one under-five child, all children would be tested and treated for malaria if the rapid diagnostic test (RDT) was positive, but a researcher would flip a coin to randomly determine which child would be enrolled in the study. No study households had more than two age-eligible children present at the time of the interview.

### Ethical treatment of human subjects

Recruitment and enrolment was done by the research team, which included a Malawian nurse and translator who received training in confidentiality and research ethics. Due to widespread illiteracy, the study team approached households with verbal informed consent at the time of recruitment, and used thumbprints to indicate consent of illiterate guardians. Copies of the informed consent were available in Chichewa (the dominant local language) and in English for any participant who requested one. This study received ethical approval from the University of Michigan Health Sciences and Behavioral Sciences Institutional Review Board and the University of Malawi College of Medicine Institutional Review Board.

### Study design and methods

All households with a child aged four months to five years were eligible for enrolment in the study. A Malawian nurse administered the questionnaire to a parent or guardian of each enrolled child in every participating household. The questionnaire included items on the child’s recent history of and treatment for malarial illness, as well as bed net ownership, insecticide treatment, and use. When the questionnaire respondent was the child’s mother, the nurse asked additional questions on the mother’s malaria illness, treatment and prevention methods during either her most recent or current pregnancy. After completing the questionnaire, and with parental consent, the nurse tested all children between four months and five years of age for malaria using SD Bioline Ag. Pf. RDT, and results were recorded on the questionnaire form. Children who had a positive RDT results were designated as cases, and those with negative RDT results were considered controls. The nurse administered one weight- and age-appropriate dose of artemether/lumefantrine combination therapy to children with positive RDT results, and provided necessary medication and instructions to parents for subsequent doses.

While the nurse and a research team member administered the questionnaire and RDTs, two researchers conducted environmental data collection with the assistance of a Malawian translator who was familiar with local agriculture. Each participating household's location (latitude, longitude and elevation) was recorded after averaging a minimum of ten readings by a hand-held Garmin eTrek Venture HC GPS unit to ensure estimated measurement error of <5 m. House structure and materials for windows, roof, and walls were visually characterized. Using the GPS waypoint for the participating house, a member of the study team (LRT) assessed land use/land cover at ≤25 m and 26–50 m of the dwelling, treating the distances from 0–25 m and 26–50 m as discrete distance bands encircling the house. In addition, surveyors counted the number of neighbouring houses within each distance band, as there was often at least one house within 50 m of a participant’s house. Four distinct types of land use/land cover were recorded: bare earth, natural vegetation/fallow land, agriculture, and brickmaking that created large mud pits. Environmental surveyors were blinded as to the malaria status of the enrolled children, and were required to agree about location and types of land cover classification before recording information. Where multiple types of land cover were present, surveyors recorded each of the types. In addition to land cover and household characteristics, the number of neighbouring households present in each band was also recorded. The Malawian translator assisted with distinguishing between actively cultivated crops and harvested/fallow cropland; however, there were no data collected on specific types of crops (e.g., maize, rice or bananas).

### Analytical methods

The outcome variable of interest was case or non-case status as defined by the dichotomous RDT result. Exposure variables include demographic information, bed net ownership and use, household building materials, and proximity to different types of land cover. Because the aims of this study pertained more to the effects of proximity to land cover at a finer scale than is generally assessed and also wished to minimize the number of neighbouring houses within the radius of a given participant’s house, land cover within a 25 m radius was the primary exposure of interest, but results were also calculated for the association of land cover between 25–50 m, and for the entire range of 0–50 m. Bivariate analyses were performed using simple logistic regression, the relevant Wald *X*^2^ and p-value. Variables found to be significantly associated with RDT status at p <0.10 were included in multivariate logistic regression (MLR). These same variables were then analysed in a generalized linear mixed model, specifically multilevel, multivariate, logistic regression (MLLR) using PROC GLIMMIX, which allows for generalized linear mixed models, including random intercepts to account for potentially correlated data due to cluster sampling strategy. As cluster selection was done for geographic units (i.e., TA and village), random intercepts for these variables were included in MLLR analysis to account for group-level effects, which would likely include many unmeasured spatially mediated environmental exposures. For further confirmation that spatial autocorrelation was accounted for in the models, each model was run with the Getis-Ord Gi* z-scores as the dependent factor. All analyses were conducted using SAS 9.2.

Spatial patterns were explored through ArcGIS 10.0. Geographic data such as elevation, rivers and administrative boundaries, were provided by the Malawi National Statistical Office. GPS point data of household locations were uploaded from the Garmin eTrex Venture HC using a GPS software application (Minnesota Department of Natural Resources). Tabular data were joined to GPS data, and all data were projected into the UTM Zone 36S projection. Clustering of cases and exposure variables was assessed using the local Getis-Ord G_i_* statistic in ArcGIS. Clustering of case and exposure variables was investigated at the level of the entire study area for each TA, and for each village, to assess scale-dependency of effects. In order to assess the role of geographic-dependence on cluster measurements, Pearson residuals from MLLR were joined to GPS locations in ArcGIS, and assessed for clustering with the G_i_* statistic.

## Results

A total of 390 children and households were enrolled, with one child ultimately being excluded from analysis because of missing RDT status, thus analysis was conducted on 389 children (Table 1). The number of enrolled children per village ranged from 16 to 98. Enrolled children had a mean age of 30.2 (SD =15.2) months with a range of four to 59 months. Of these 389 children, 44.5% (n = 173) were RDT positive for *Plasmodium* infection (“cases”). Additional study participant characteristics are in Table 1. The distance between participant households varied among villages, ranging from 29.75 to 82.67 m. The mean distance between participants’ houses for all villages was 68.0 m in TA Sitola and 61.9 m in Nsamala, but these means were not significantly different from one another (p = 0.68).

**Table 1:** Demographic and household physical characteristics of 390 study participants surveyed during June to August 2011 residing in ten villages from two Traditional Authority locations in the Machinga District Hospital catchment area of southern Malawi

**Participant and household characteristics**	**n (%)**	**Participant and household characteristics**	**n (%)**
**Traditional Authority (TA)**		**Wall building material**	
Sitola	161 (41.39)	Mud (sun-dried bricks)	249 (64.34)
Nsamala	228 (58.61)	Brick (fired bricks)	130 (33.59)
**Villages**		Cement	6 (1.55)
*Sitola*		Other	2 (0.52)
Lawrence	25 (6.43)	**Windows**	
Tambula	33 (8.48)	No windows	130 (33.68)
Nyama	33 (8.48)	Open windows	124 (32.12)
Mboma	20 (5.14)	Glass windows	92 (23.83)
Mbaya	34 (8.74)	Screened windows	7 (1.81)
Botoni	16 (4.11)	Other	33 (8.55)
*Nsamala*		**Agriculture 0–25 m from house**	
Ntonya	27 (6.94)	Yes	78 (20.05)
Chilembwe	98 (25.19)	No	311 (79.95)
Sitima	66 (16.97)	**Agriculture 25–50 m from house**	
Kaniche	37 (9.51)	Yes	105 (26.99)
**Sex of child**		No	284 (73.01)
Male	205 (52.70)	**Agriculture 0–50 m from house**	
Female	184 (47.30)	Yes	115 (29.5)
**Household owned ≥1 bed net**		No	274 (70.44)
Yes	207 (53.35)		
No	181 (46.65)		
**Malaria symptoms in past 2 weeks**			Mean (sd)*
Yes	132 (34.11)	**Age of child (months)**	30.13 (15.19)
No	255 (65.89)	**Household elevation (m)**	524.59 (37.91)
**RDT result**		**Number of people living in household**	4.36 (1.61)
Positive	173 (44.47)	**Number of neighbouring houses <25 m**	1.15 (1.18)
Negative	216 (55.53)	**Number of neighbouring houses 25–50 m**	1.54 (1.55)
**Roofing materials**		**Total number of neighbouring houses**	2.77 (2.14)
Thatch	322 (83.20)		
Metal	65 (16.80)		

* - standard deviation.

From bivariate Chi-square analysis, cases were found to be 4.2 times more likely than non-cases to live in TA Sitola than TA Nsamala (95% CI 2.74-6.46), and were 2.9 times more likely to live within 25 m of active dry-season agriculture crops (95% CI 1.74-4.92) (Table 2). In addition to geographic and environmental differences, cases and controls differed significantly by age, sex, bed net ownership, and house roofing materials (Table 2). Houses within TA Sitola were 9.0 times more likely to have agriculture within 25 m (95% CI 4.90-16.66), and be situated at an average elevation that was 54.1 m higher than houses in Nsamala (p <0.0001). Other differences between children residing in either of the TAs included age, sex, and likelihood of bed net ownership (Table 3). All hypothesized exposure variables significant at p < 0.10 in bivariate analysis were included in MLR models. Interaction terms for highly correlated exposure variables were also included in MLR models.

**Table 2:** **Univariate comparison of RDT-based *Plasmodium *** **infection status (case, control) and demographic and household environmental characteristics among study participants**

**Participant and household characteristics**	**Cases**	**Controls**	**Wald**	**p-value**	**OR**	**Wald**
	**n (%)**	**n (%)**	**×**^**2**^			**95% CI**
**Study sample**	173 (44.47)	216 (55.53)	4.75	**0.03**		
**Sex of child**						
Female	98 (25.19)	86 (22.11)	10.92	**0.001**	1.98	1.32-2.96
Male (ref.)	75 (19.28)	130 (33.42)			1.00	
**Traditional Authority**						
Sitola	104 (26.74)	57 (14.65)	43.02	**<0.0001**	4.20	2.74-6.46
Nsamala (ref.)	69 (17.74)	159 (40.87)			1.00	
**Reported malaria in <2 weeks**						
Yes	83 (21.45)	49 (12.66)	26.77	**<0.0001**	3.11	2.01-4.81
No (ref.)	90 (23.26)	165 (42.64)			1.00	
**Owned at least one bed net**						
No	99 (25.52)	82 (21.13)	14.03	**0.0002**	0.46	0.31-0.69
Yes (ref.)	74 (19.07)	133 (34.28)			1.00	
**Wall materials**						
Mud	102 (26.49)	147 (38.18)	3.40	0.07	1.48	0.97-2.26
Brick/cement (ref.)	69 (17.92)	67 (17.40)			1.00	
**Roofing materials**						
Thatch	151 (39.02)	171 (44.19)	5.70	**0.02**	0.50	0.28-0.89
Metal (ref.)	20 (5.17)	45 (11.63)			1.00	
**Windows**						
Glass	38 (9.84)	54 (13.99)	0.52	0.47	0.84	0.52-1.35
Other (ref.)	134 (34.72)	160 (41.45)			1.00	
**Agriculture <25 m from house**						
Yes	51 (13.11)	27 (6.94)	17.28	**<0.0001**	2.93	1.74-4.92
No (ref.)	122 (31.36)	189 (48.59)			1.00	
**Agriculture 25–50 m from house**						
Yes	62 (15.94)	43 (11.05)	12.37	**0.0004**	2.25	1.42-3.55
No (ref.)	111 (28.53)	173 (44.47)			1.00	
**Agriculture 0–50 m from house**						
Yes	67 (17.22)	48 (12.34	12.57	**0.0004**	2.21	1.42-3.45
No (ref.)	106 (27.25)	168 (43.19)			1.00	
House elevation* (per 10 m)			4.92	**0.02**	1.06	1.01-1.11
Age of child* (months)			16.46	**<0.0001**	1.03	1.02-1.04
Number of people within household*			2.41	0.12	1.10	0.97-1.25
Number of houses within 25 m*			1.82	0.18	0.89	0.75-1.06
Number of houses within 25–50 m*			0.11	0.74	0.98	0.86-1.11
Total neighboring houses 0–50 m*			0.73	0.39	0.96	0.875-1.05

*- continuous variables.

**Table 3:** Characteristics of study participants by Traditional Authority of residence (Sitola or Nsamala) in southern Malawi surveyed during June to August 2011

**Participant and household characteristics**	**Sitola**	**Nsamala**	**×**^**2**^	**p-value**	**OR**	**95% CI**
	**n (%)**	**n (%)**				
**Study sample**	161 (41.39)	228 (58.61)	11.54	**<0.001**		161 (41.39)
**Sex of child**						
Female	85 (21.85)	99 (25.45)	3.33	0.07	1.46	85 (21.85)
Male (ref.)	76 (19.54)	129 (33.16)			1.00	76 (19.54)
**Traditional Authority**						
Sitola	104 (26.74)	69 (17.74)	45.04	**<0.0001**	4.20	104 (26.74)
Nsamala (ref.)	57 (14.65)	159 (40.87)			1.00	57 (14.65)
**Reported malaria in <2 weeks**						
Yes	70 (18.09)	62 (16.02)	11.81	**0.0006**	2.11	70 (18.09)
No (ref.)	89 (23.00)	166 (42.89)			1.00	89 (23.00)
**Owned at least one bed net**						
No	87 (22.42)	94 (24.23)	6.53	**0.011**	0.59	87 (22.42)
Yes (ref.)	73 (18.81)	134 (34.54)			1.00	73 (18.81)
**Wall materials**						
Mud	152 (39.48)	75 (19.48)	1.26	0.26	1.27	152 (39.48)
Brick/cement (ref.)	97 (25.19)	61 (15.84)			1.00	97 (25.19)
**Roofing materials**						
Thatch	138 (35.66)	184 (47.55)	1.81	0.23	0.68	138 (35.66)
Metal (ref.)	22 (5.68)	43 (11.11)			1.00	22 (5.68)
**Windows**						
Glass	38 (9.84)	54 (13.99)	0.007	0.93	1.02	38 (9.84)
Other (ref.)	120 (31.09)	174 (45.08)			1.00	120 (31.09)
**Agriculture <25 m from house**						
Yes	63 (16.20)	15 (3.86)	62.37	**<0.0001**	9.12	63 (16.20)
No (ref.)	98 (25.19)	213 (54.76)			1.00	98 (25.19)
**Agriculture 25–50 m from house**						
Yes	79 (20.31)	26 (6.68)	67.93	**<0.0001**	7.49	79 (20.31)
No (ref.)	82 (21.08)	202 (51.93)			1.00	82 (21.08)
**Agriculture 0–50 m from house**						
Yes	86 (22.11)	29 (7.46)	75.06	**<0.0001**	7.87	86 (22.11)
No (ref.)	75 (19.28)	199 (51.16)			1.00	75 (19.28)
**Continuous variables**	**Sitola (mean)**	**Nsamala (mean)**	**t-value**	**p > t**		
**House elevation** (per 10 m)	556.3	502.2	−19.93	**<0.0001**		
**Age of child** (months)	29.53	30.64	0.69	0.49		
**Number of people within household**	4.33	4.37	0.26	0.79		
**Number of houses within 25 m**	1.11	1.18	0.58	0.56		
**Number of houses within 25–50 m**	1.48	1.57	0.54	0.59		
**Total neighboring houses 0–50 m**	2.78	2.75	−0.17	0.86		

Using MLR, the associations between positive RDT status and child’s age, sex, bed net ownership, proximity to agriculture (<25 m), household elevation (m), roofing, and wall materials were evaluated (Table 4). Because of concerns about geographic dependence of some variables, this analysis was followed with MLLR using PROC GLIMMIX. By including random effects intercepts for TA and village, some estimates of relative risk changed, but results were broadly consistent with simple MLR results (Table 4). The strength of the association between close household proximity to agriculture and *Plasmodium* infection remained relatively unchanged after expanding analysis from crude bivariate (OR = 2.93) to multilevel, multivariate logistic regression (OR = 2.80). This similarly greater risk of infection near active dry-season agriculture was observed even after having controlled for bed net ownership.

**Table 4:** **Odds ratios of statistically significant risk factors for being *Plasmodium *** **positive (case) based on simple logistic regression (SLR) and multilevel logistic regression (MLLR) models**

	**SLR model**	**MLLR model**
	**OR (95% CI)**	**OR (95% CI)**
**Agriculture <25 m**	**3.02 (1.69-5.39)**	**2.78 (1.39-5.55)**
**Elevation (per 10 m)**	1.00 (0.97-1.09)	**0.82 (0.68-0.98)**
**Child age (years)**	**1.46 (1.23-1.74)**	**1.52 (1.25-1.85)**
**Child being female**	**1.99 (1.28-3.10)**	**1.93 (1.19-3.15)**
**Bed net ownership**	**0.47 (0.30-0.72)**	**0.54 (0.33-0.90)**

**Figure 2:**
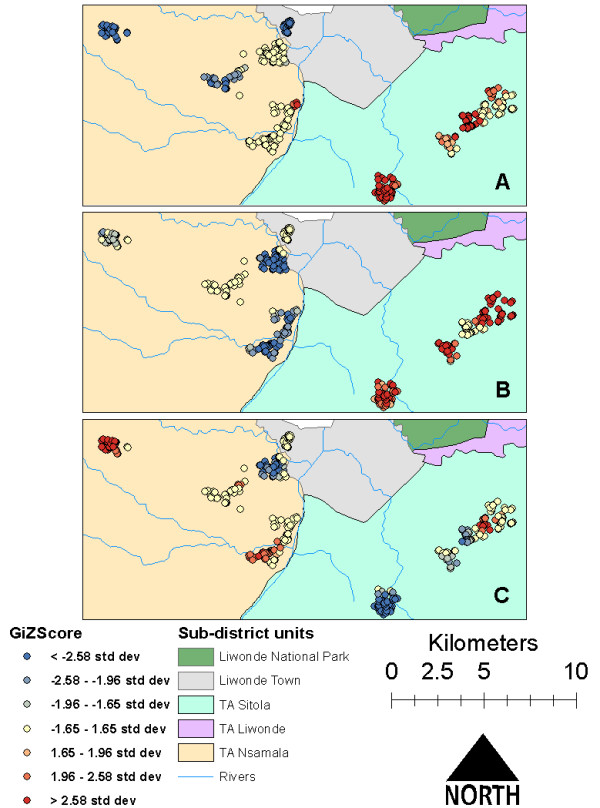
Univariate Gi* hotspots of A) malaria cases, B) houses <25 m from agriculture, and, C) bed net ownership in 390 households from ten villages near Liwonde, Malawi during June to August 2011.

Univariate spatial analysis of clustering and dispersal was conducted using the Getis-Ord G_i_* for all significant variables in bivariate statistical analysis. While the distributions of positive household clusters and of households within 25 m of agriculture overlap significantly, positive and negative clusters of bed net ownership were more widely distributed (Figure 2). Although more households in TA Nsamala than TA Sitola owned bed nets, household proximity to agriculture remained strongly predictive of child malaria status after controlling for bed net ownership. Unlike the Gi* hot/cold spot maps for cases and households within 25 m of agriculture, the distribution of bed net ownership did not exclusively cluster in TA Nsamala which had lower *Plasmodium* infection prevalence (Figure 2(c)). Instead, bed net hotspots were found in both TAs, although more so in Nsamala than Sitola.

**Figure 3:**
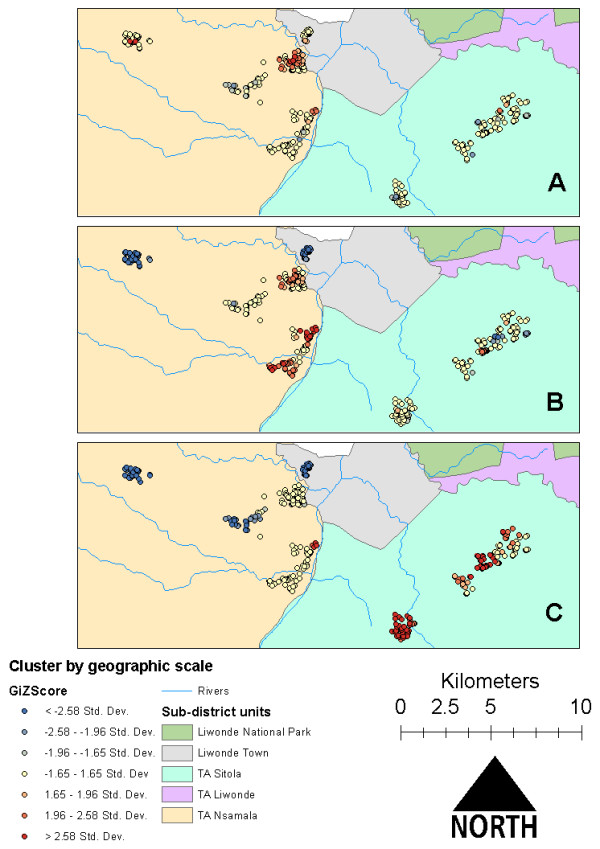
***Plasmodium *** **infection case clusters assessed at the scales of A) village, B) Traditional Authority (TA), and, C) entire study area extent near Liwonde, Malawi during June to August 2011.**

**Figure 4:**
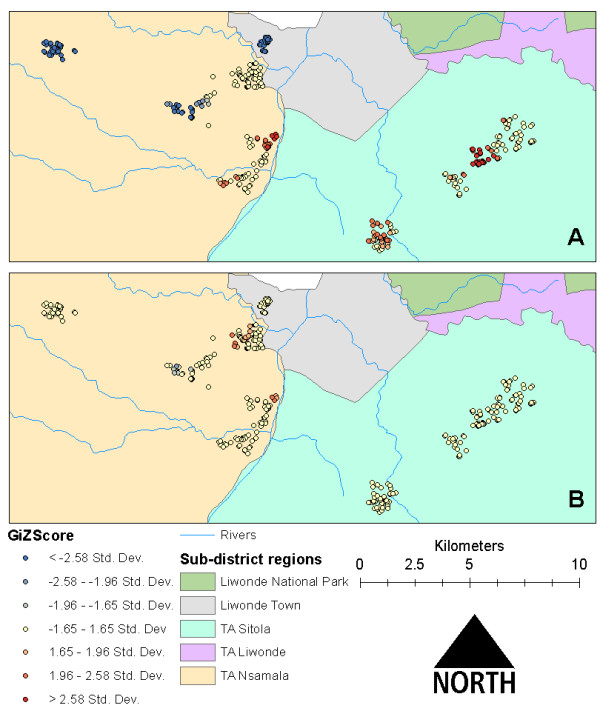
**Comparison of residual clustering of *Plasmodium *** **infection of children in households near Liwonde, Malawi based on A) simple logistic regression and B) multilevel logistic regression models.**

Assessment of case clustering at the level of village, TA and study extent revealed remarkably different patterns of negative and positive clusters among the different levels. For example, at the village level there was almost no clustering of cases or controls, while “hot spots” and “cold spots” were in nearly opposite locations in TA level and full study, extent level analyses (Figure 3). Pearson residuals from MLR linked to GPS points of participant households exhibited a similar distribution of clusters in TA Sitola, with some extension of the risk map into TA Nsamala. Residuals from MLLR showed very little evidence of clustering, which suggests that much of the unmeasured spatial, social and environmental variation was accounted for with the random effects intercepts of MLLR (Figure 4).

## Discussion

Results from both multivariate and spatial statistical analyses indicate that dry-season malaria risk in children is determined by fine scale, geographic/environmental factors, particularly household level, peridomestic crop production. In both bivariate and multivariate statistical analysis, children who lived in TA Sitola had much higher odds of testing positive for malaria and living near an active agricultural site when compared to children in nearby TA Nsamala. Spatial statistical analysis confirmed the multivariable results, with significant clusters of cases occurring in TA Sitola, and significant clusters of non-cases in TA Nsamala.

Given the cluster sampling strategy, there was concern that clustering of cases and of risk factors might have been an artifact of that selection process, thus violating the assumption of independence. MLLR, however, addressed this potential source of bias. Interestingly, when the association between RDT status and bed net ownership was stratified by TA, the results indicated that bed net ownership had no effect on malaria status in Nsamala, but was significantly protective in Sitola. By considering this observation along with the location of agricultural clusters in Figure 2(b), there may be underlying additional, unmeasured environmental/ecological factors affecting malaria transmission in Sitola that were not present in Nsamala.

Mapping the residuals of both MLR and MLLR in ArcGIS allowed assessment of the degree of variation in data explained by the modelled variables, while controlling for unmeasured geographic features and sampling strategy. While univariate cluster analysis in GIS showed discrete clusters of malaria cases and households with proximity to agriculture in TA Sitola (Figure 2(a) and (b)), the MLR Pearson residual map (Figure 4(a)) extends the malaria-positive (high value) clusters into TA Nsamala. This expansion into Nsamala is consistent with observed ecological patterns, as those villages in Nsamala that are nearest Sitola also border the Shire River. After including random effects estimates for TA and village in the MLLR, almost all variation in TA Sitola was explained, as evidenced by the lack of any significant clustering of Pearson residuals in that region (Figure 4(b)). Interestingly, there are some indications that factors other than those included in the model had a significant relationship with *Plasmodium* prevalence in TA Nsamala, while risk was drastically lower than in Sitola. There were some high value clusters along the Shire River in Nsamala, where other unmeasured variables may modify local dry-season malaria risk beyond those related to age, elevation, bed net ownership and proximity to active agriculture. This suggests that, while multilevel modelling was able to account for some unmeasured spatial and environmental variation, this method may be less useful in low-prevalence/low-risk settings, where it may still be necessary to directly measure variables of interest.

The scale-dependence of clusters shown in Figure 3 can be explained in light of this residual mapping. When assessed at the village level, there is insufficient environmental variation to affect transmission locally. At the TA level, cases clustered in TA Nsamala along the river, which is reflected in the MLR residual map, while almost no clusters appeared in TA Sitola. This suggests that malaria risk is more uniformly distributed across Sitola than in Nsamala, which could be a result of more widespread ecological conditions conducive to increased vector abundance in Sitola. The relationship between dry-season, active agriculture and malaria may be explained by underlying regional ecological conditions that are conducive both to extended agricultural seasons and *Anopheles* mosquito reproduction/survival. Those conditions could include relatively high levels of soil moisture, local humidity, temperature, and standing water, all of which should enhance anopheline survival and reproduction [27-29]. As one example, the lack of actively cultivated crop land in Nsamala suggests that even relatively hardy crops such as cassava are unable to be widely grown there during the dry season, which could be due to a number of unmeasured environmental or social factors, such as the ability of soil to retain moisture [30,31], differences in rainfall associated with elevation in hilly regions [32], or reduced environmental capacity or social ability to sustain irrigation schemes [33].

The results of this study are consistent with the findings of other studies that examined the relationship between fine-scale land cover heterogeneity or smallholder agriculture on malaria. Indoor microclimates varied significantly depending on whether houses were located in deforested or naturally forested areas over a relatively compact 4 sq km study area, and relatively small fluctuations in mean indoor temperature shortened the duration of mosquitoes’ first and second gonotrophic cycles [28], which suggests one potential means by which fine-scaled heterogeneity in land cover might affect malaria transmission. The effect of converting swamp to agricultural land was similarly shown to increase local village evening minimum temperature, which would promote increased mosquito population size and longevity while shortening mosquito gonotrophic cycle duration and parasite extrinsic incubation period [33]. These two studies suggest that local-scale development of agriculture can create conditions favourable to mosquito reproduction and longevity by affecting village-level temperature. The relationship between malaria and classified types of agricultural land cover within 2 km of villages showed a great deal of variation based on crop type and proximity, with weaker associations at shorter distances [17]. However, even the shortest distance band used in that study would mask intravillage variation in malaria risk attributable to even closer proximity to high-risk crop types, as there would be higher expected levels of convergence of exposures within a larger distance band.

Despite being cross-sectional and case–control in design, this study has many strengths. A relatively large sample size was recruited across a range of villages of different sizes and geographic settings. All sampled households were in one public hospital catchment area, which provided similar levels of access to free medical services. By administering a questionnaire in conjunction with environmental field survey, multiple possible contributors to malaria risk could be examined in this setting. Multilevel, multivariate logistic regression with cluster analysis in GIS allowed for assessment of risk factors and accounted for the cluster sampling strategy that has rarely been considered in such epidemiological field studies. With direct survey of land cover, data were collected on a finer geographic scales than are available through many remote-sensing applications.

Unfortunately, information was not obtained on the size and nature of agricultural sites (e g, type of crop), nor were entomological parameters related to vector mosquitoes or people’s socio-economic status directly measured. Future research in the area should include these variables. Recent, fine-scale, satellite image data on land use/land cover were also not available. Despite the use of point averaging of household locations, GPS measurements are likely to be accurate to within only 5 m. However, even under circumstances of maximum measurement error, the land cover data remain capable of assessing differences in household-level vegetation patterns. It is further possible that land covers was incorrectly categorized in some instances, however, such errors would likely reduce the strength of observed associations, and would not be biased since researchers assessing land cover were blinded to the RDT status of enrolled children. Thus, these rigorously analysed observations can be said to accurately reflect a surprisingly strong association between dry-season cropping nearby households and malaria risk among residents. This association was observed using different analytic methods and after controlling for possible confounders. Another potential limitation of this study is the reliance on RDT for diagnosis of recent malaria, which, although highly sensitive, have been shown to have low specificity in comparison to microscopy or PCR-based diagnostics [3,34]. However, since the primary outcome of interest in this study was recent infection as opposed to parasitaemia, this limitation should not greatly affect interpretation of these results.

## Conclusion

This study examined the relationships between childhood malaria and household proximity to directly observed, active, dry-season crop production on a scale of <25 m, finer than that of most previous studies. Analysis demonstrated that proximity to agriculture increases the odds of malaria in children under-five by nearly three-fold, even after controlling for age, sex, household elevation, and bed net ownership. The combination of multilevel statistical analysis and spatial analysis in GIS provides a powerful means to describe and analyse environmental risk patterns for malaria. These results suggest that measuring disease transmission parameters may be complicated in spatially heterogeneous regions, and that inferences may depend in important ways on the sampling scale. Since dry seasons are normally considered periods of relatively low malaria incidence, it is worth noting that in some villages *Plasmodium* infection prevalence was still at >50%. This type of fine-scale modelling of malaria risk allows for more efficient targeting of prevention and intervention programmes. Future applications of these methods could integrate the molecular biology of mosquito vectors and other entomological variables across fine spatial scales, along with high resolution remote sensing data of land cover and other ecological and environmental parameters. Such information may be helpful in understanding both the patterns of risk at scales where transmission is occurring, and the underlying mechanisms that are giving rise to that risk.

## Competing interests

The authors declare that they have no competing interests.

## Authors’ contributions

LRT conceived of the study, participated in study design, collected environmental and survey data, performed the statistical and spatial analyses, and drafted the manuscript. DM participated in study design and coordination, and revised the draft manuscript. DPM participated in study design and critically revised the manuscript. MLW participated in study design, and helped draft the manuscript. All authors read and approved the final manuscript.
